# In vivo assay for the evaluation of the effect of anesthesia on locomotor activity in the weakly electric fish *Apteronotus leptorhynchus*

**DOI:** 10.1007/s10695-025-01546-3

**Published:** 2025-08-11

**Authors:** Mariam Ahmed, Günther K. H. Zupanc

**Affiliations:** Laboratory of Neurobiology, Department of Biology, Northeastern University, Boston, MA 02115 USA

**Keywords:** Neuro-Behavioral Assay, Anesthetics, MS-222, Locomotor activity, Electric organ discharge, *Apteronotus leptorhynchus*

## Abstract

Despite a nearly 200-year long history of anesthesiology, there is a persistent need for new anesthetics with improved safety and specificity, both in animals and humans. The identification and characterization of candidate molecules for the development of such novel drugs depends crucially on suitable in vivo assays. Ideally, these assays test for gradual behavioral outcomes and for the possibility of differential effects on different behaviors and/or neural functions induced by an anesthetic agent. To accommodate these features, we have developed an in vivo assay, based on analysis of amplitude modulations of the electric organ discharge of the weakly electric fish *Apteronotus leptorhynchus*. It provides a quantitative measurement of the gradual changes in locomotor activity induced by anesthetics. An extension of this assay offers the opportunity for simultaneous evaluation of the effects of anesthetics on two neural functions of the brainstem oscillator that controls the fish’s electric behavior. As a distinctive, unique feature, this multimodal Neuro-Behavioral Assay collects all data utilized for analysis from a single non-invasive recording of the electric organ discharge of the fish.

**Supplementary Information:**

The online version contains supplementary material available at 10.1007/s10695-025-01546-3.

## Introduction

Despite a nearly 200-year long history of anesthesiology (for review see Robinson and Toledo 2012), there is a persistent need for new anesthetics with improved safety and specificity, both in animals and humans. The development of new anesthetics has been grounded largely on chemical modification of the molecular scaffold, or on reformulation, of existing drugs. For instance, fospropofol is a water-soluble prodrug of the most widely used lipophilic anesthetic, propofol (for review see Feng et al. 2017). More recent attempts to identify novel anesthetics have increasingly included the screening of drug libraries, based on the assumed ability of these compounds to modulate the function of established molecular targets, such as activation of γ-aminobutyric acid type A (GABA_A_) receptors (Jenkins et al. 2001; Reynolds et al. 2003; Joesch et al. 2008; Liu et al. 2008; Krasowski and Hopfinger 2011). Naturally, however, this approach prohibits the detection of anesthetics that act via other molecular targets. Thus, identification of such novel anesthetics requires a different, mechanism-independent screening approach.

Most commonly, experimental tools based on the latter screening approach test for behavioral deficits induced by anesthetics. Such deficits include the loss of righting reflexes in laboratory mice (*Mus musculus*) (Sun et al. 2006) as well as in tadpoles and adults of American bullfrogs (*Rana catesbeiana*) and African clawed frogs (*Xenopus laevis*) (Downes and Courogen 1996; Krasowski et al. 2001); the lack of startle response in *X. laevis* tadpoles (McKinstry-Wu et al. 2015); and the changes in photomotor response in zebrafish (*Danio rerio*) larvae (Yang et al. 2018).

A property shared by each of these assays is a binary behavioral outcome, e.g., the presence vs. absence of a reflex response. This simplifies the monitoring of the behavior and facilitates the use of these assays for high-throughput screening. Yet, a major disadvantage is that only behavioral endpoints, but not graded responses, are available for assessment, although both the acquisition of different stages and planes of anesthesia induced by the anesthetic(s) and the subsequent recovery from the state of anesthesia are characterized by gradual transitions. Moreover, none of these assays offers the option to evaluate the neural effects of a given anesthetic on central nervous system (CNS) functions other than the selected motor response. However, the assessment of multiple CNS functions offers a significant advantage by providing a more comprehensive picture of on-target and off-target effects of anesthetic compounds.

**Fig. 1:**
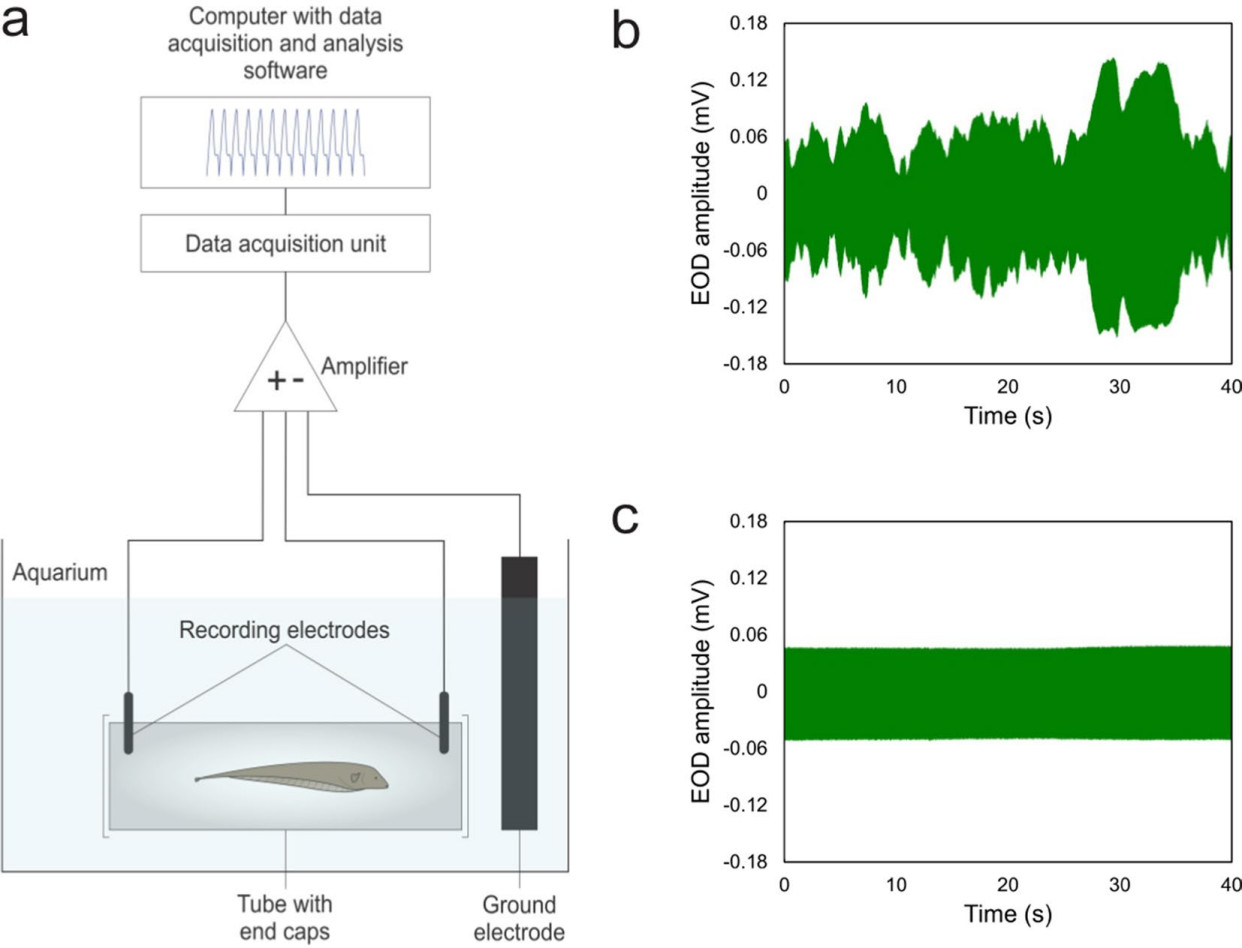
Experimental setup for non-invasive recording of the EOD (**a**). During the experiment, the fish is restrained to an opaque cylindrical shelter tube by closing the two open ends with a coarse plastic mesh netting. The differential-recording electrodes, made of stainless-steel rods, are mounted on the inside of the tube. The amplified electric signal is fed into an analog-to-digital converter for computer software-assisted analysis. Variation of the fish’s position relative to the stationary recording electrodes leads to amplitude modulations of the EOD. The two time–voltage graphs show 40-s EOD traces from the same fish recorded during high locomotor activity (**b**) and extremely low locomotor activity induced by anesthesia (**c**). (Fig. 1a from Eske et al. 2023.)

To address the latter two issues, we have, in the present study, developed an in vivo assay that enables investigators to quantify the gradual reduction of locomotor activity induced by an anesthetic, and the subsequent recovery of this behavior, on a continuous scale. This assay is based on the analysis of amplitude modulations of the electric discharges of the brown ghost knifefish (*Apteronotus leptorhynchus*). The fish’s electric dipole field is recorded by stationary external electrodes (Fig. 1a). Whereas the amplitude of the *emitted* discharges is stable, any movements of the fish that change its distance and/or orientation to these electrodes will be registered by modulations of the *recorded* discharges (Fig. 1b).

Besides this property of the recorded EOD, the in vivo assay (in the following referred to as Neuro-Behavioral Assay) offers the opportunity to combine the quantitative analysis of the locomotor behavior with the simultaneous evaluation of the effect of anesthesia on two additional behavioral outputs. These two readouts are changes in the frequency of the electric organ discharge (EOD) and in the rate of transient frequency/amplitude modulations (‘chirps’) of the EOD. Both behaviors have been extensively studied in terms of their functions and underlying neural control (for reviews see Zupanc and Bullock 2005; Zupanc 2018) and occur both spontaneously and during social interactions. Yet, transient changes in EOD frequency evoked by a conspecific are rare and restricted to a few Hertz, whereas all anesthetics examined thus far reliably induce decreases in EOD frequency that may involve frequency drops larger than 100 Hz (Eske et al. 2023; Lehotzky et al. 2023). Chirps are very rarely produced spontaneously, but their rate may increase significantly when the fish perceives the electric field of a conspecific or a mimic thereof (Engler and Zupanc 2001). During social interactions, out of a total of 6 chirp types, predominantly chirps of type 2 are produced, which are characterized by a short duration (approximately 20 ms), a maximum frequency increase not exceeding 100 Hz on average, and a slight reduction in amplitude (Engler and Zupanc 2001; Zupanc et al. 2006). Type-2 chirps are also the most frequently generated chirp type induced by anesthetics (Eske et al. 2023; Lehotzky et al. 2023).

As a distinctive feature of the resulting trimodal assay, the data used for analysis of all three behavioral outputs are collected from a single non-invasive recording of the electric discharges of the fish and can, thus, be directly compared. Moreover, each of the three behavioral readouts is provided on a continuous scale. These unique features enable investigators to characterize the behavioral effects of anesthetic compounds with superior quality, compared to traditional single-modal assays that are restricted to the analysis of a binary behavioral outcome.

## Materials and methods

### Animals

A total of 8 *A. leptorhynchus*, obtained from their natural habitat in Colombia through a tropical fish importer (Segrest Farms, Gibsonton, Florida, USA), were used in this study. They were approximately 3 years old. Based on a sexual dimorphism in EOD frequency (Zupanc et al. 2014), the 5 fish (Fish 01, 07, 08, 09) with the highest frequencies were identified as males, whereas the 3 fish (Fish 04, 11, 12) with the lowest frequencies were females. One fish (Fish 10) could not be unambiguously sexed because it emitted EODs within a frequency band shared by both males and females.

The fish had been kept in individual tanks (50 cm × 30 cm × 30 cm), in the following referred to as ‘home tanks,’ for about 2 years. These tanks were equipped with aquarium thermostat heaters and air-driven corner filters. The aquarium water was prepared by adding a mixture of inorganic salts (81 mmol/L MgSO_4_·7H_2_O; 107 mmol/L KCl; 12 mmol/L NaH_2_PO_4_·2H_2_O; 732 mmol/L CaSO_4_·2H_2_O) to deionized water until a water conductivity of approximately 200 μS/cm was reached. The pH varied between 7.7 and 7.9, and the water temperature ranged between 26 °C and 28 °C. A 12:12 h light:dark photoperiod was maintained with a timer. All experiments were conducted during the light phase. The fish were fed red mosquito larvae daily, after all experiments for that day had been completed.

In each tank, an opaque cylindrical plastic tube (length: 190 mm; inner diameter: 38 mm; outer diameter: 42 mm) provided shelter and was readily accepted by the fish, particularly during the light phase, when individuals of this nocturnal species spend most of the time in shelter places (Fig. 1a). A pair of stainless-steel electrodes was mounted on the inside of each tube (see ‘EOD recording,’ below).

### EOD recording

Differential recording of the fish’s EOD was done through the pair of stainless-steel electrodes built into the shelter tube (Fig. 1a). During the experiment, the two open ends of the shelter tube were closed with a coarse plastic mesh netting to ensure that the fish did not leave the tube. This allowed the fish’s EOD to be recorded continuously without interruptions. At the same time, mesh size was chosen such that sustained water flow through the tube was ensured. A strip of stainless steel was placed in the tank to serve as a ground electrode.

The signal was AC amplified (gain: 30 ×; low-pass filter: none; high-pass filter: 200 Hz) by a CED 1902 amplifier (Cambridge Electronic Design, Cambridge, England) and then digitized at a sampling rate of 50 kHz using a CED Micro 1401 mkII analog-to-digital converter (Cambridge Electronic Design), a Lenovo ThinkCentre V50s SFF desktop computer (equipped with an Intel Octa Core i7-10.700, 32 GB RAM, 512 GB NVMe + 1 TB HDD), and the software program Spike 2 Version 5.21 (Cambridge Electronic Design).

### Quantification of locomotor activity

For this study, data were collected from 35 consecutive minutes of each EOD recording, encompassing 15 min immediately preceding treatment, 5 min of treatment (anesthesia or control), and 15 min immediately following treatment. During transfer of the fish from its home tank to the experimental tank and back to the home tank, the EOD could not be recorded, and thus was not available for analysis. This caused a gap in the analysis ranging from 6–21 s (median: 10.5 s; *n*: 32 transfers, involving 4 transfers of each of the 8 fish).

Locomotor activity was assessed by quantifying the movements of the fish relative to the position of the recording electrodes in the shelter tube. To analyze the changes in the recorded EOD amplitude, the voltage data of the EOD recordings were divided into 1-s segments (each containing 50,000 samples, equivalent to the sampling rate); the peak-to-peak amplitude was determined using the *peak-2-peak* function in MATLAB version R2022a in each segment; and the standard deviation of the peak-to-peak amplitudes was calculated over 10 s. The latter was defined as Locomotor Activity Index (LAI; in mV).

### Calculation of EOD frequency

For calculation of EOD frequency, the method described by Lehotzky et al. (2023) was used. The frequencies were adjusted to a reference temperature of 26 °C, using a Q_10_ of 1.56 (Zupanc et al. 2003).

### Chirp detection

Chirps were detected using the method by Eske et al. (2023). This approach is optimized for the identification of type-2 chirps, which are predominantly produced in response to anesthesia.

### Experimental design

**Fig. 2:**
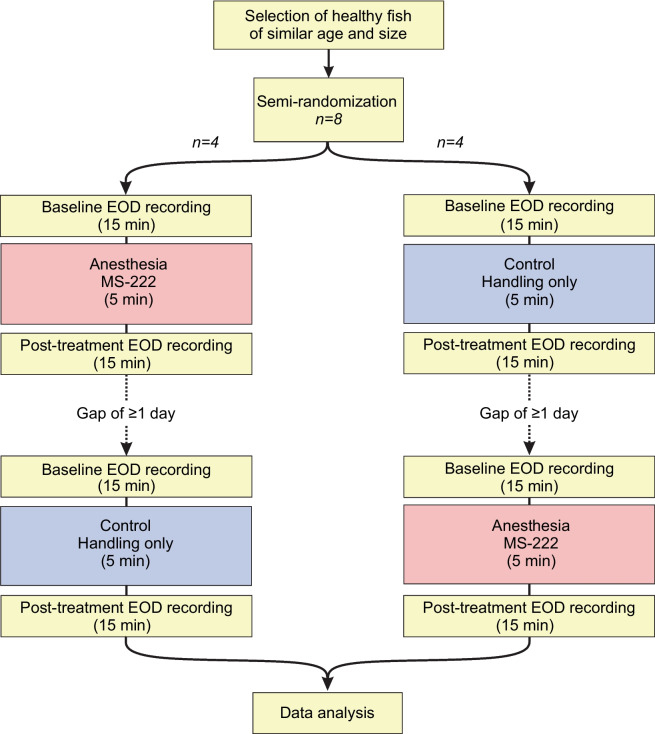
Flow diagram outlining experimental design

To examine the effect of MS-222 on locomotor activity, EOD frequency, and chirping behavior, the following experiments were conducted using 8 fish (Fig. 2). First, to establish baseline activity, the EOD of the fish was recorded in its home tank for 15 min. Then, the shelter tube with the fish was transferred to a separate, smaller tank (‘experimental tank;’ 30 cm × 20 cm × 20 cm), where the recording continued during a 5-min exposure of the fish either to 0.02% MS-222 (Western Chemical, Washington, USA) dissolved in water from the home tank (‘anesthesia experiment’) or to water from the home tank only (‘control experiment’). This concentration of MS-222 was shown previously to be effective in inducing a state of anesthesia in *A. leptorhynchus* (Eske et al. 2023; Lehotzky et al. 2023). The solution was buffered with sodium bicarbonate to a pH of approximately 7.5. The handling of the shelter tube with the fish was identical under both experimental conditions. The temperature in the experimental tank was similar to the one in the home tank (median difference of temperature home tank – temperature experimental tank: −0.05 °C; range: −0.50 to + 0.22 °C; *n*: 8 fish, each subjected to anesthesia and control condition). Finally, the fish in its shelter tube was returned to the home tank, where the EOD was recorded for another 15 min.

Each of the 8 fish was subjected to 1 anesthesia experiment and 1 control experiment. These experiments were conducted on different days, with the gap between the end of the first experiment and the beginning of the second experiments ranging from 17–262 h (median: 34 h). The order of the anesthesia experiment and the control experiment was determined using a randomized complete block design.

### Statistical analysis

Statistical analysis of the data was performed using the software package IBM SPSS Statistics Version 29.0.1.1 (IBM Corporation, Armonk, New York, USA).

### Video recording

For video recording of the fish’ locomotor activity, we used a Sony HDRCX405 HD Video Recording Handycam Camcorder (Sony Corp.) mounted on a tripod.

## Results

### Locomotor activity in the recording tube

During all experiments, EOD recordings were carried out by restraining the fish to the inside of the recording tube (Fig. 1a). There, the fish spent most of the time in a position with its anteroposterior axis parallel to the longitudinal axis of the tube, and its dorsal side pointing upward. Locomotor activity included swimming in anterior or posterior direction; turning the body around its dorsoventral axis; tilting the body sideward; and performing 360° rotations around the anteroposterior axis (see video clips shown in Supplementary Information 1). Through these various forms of locomotor activity, the fish’s relative position to the stationary recording electrodes changed, thereby resulting in amplitude modulations of the recorded EOD (Fig. 1b).

### Constancy of locomotor activity

**Fig. 3:**
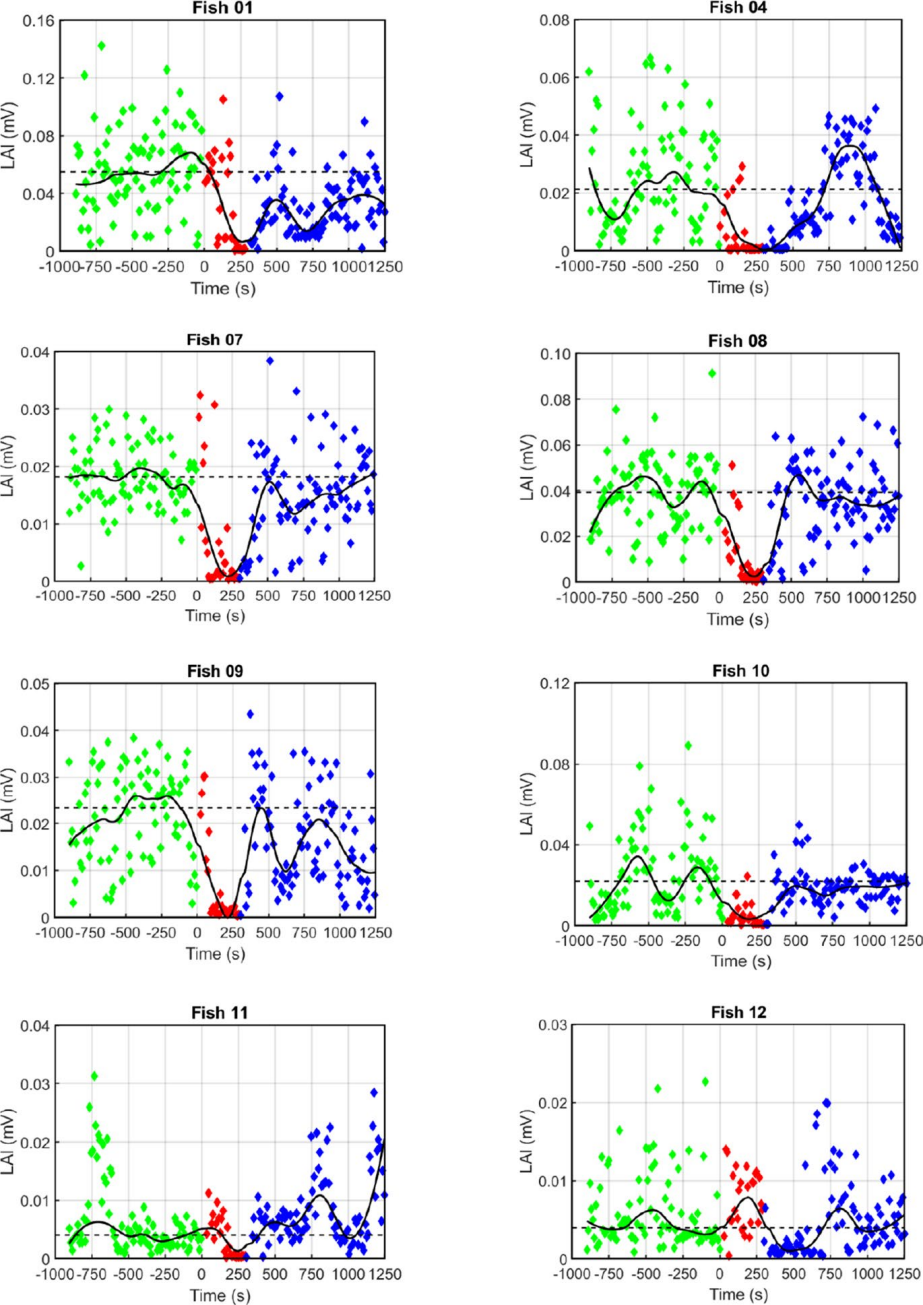
Effect of MS-222 anesthesia on locomotor activity assessed by determining the LAI every 10 s. Three time segments of the continuous EOD recording of each fish were analyzed: 15 min immediately prior to the anesthesia in the fish’s home tank (*green diamonds*); 5 min exposure of the fish to the anesthesia in the experimental tank (*red diamonds*); and 15 min immediately following the transfer of the fish from the anesthesia tank to the home tank (*blue diamonds*). The time point at which the fish was transferred to the anesthesia tank with the MS-222 solution was arbitrarily defined as ‘0.’ Since EOD measurements during the transfer were not possible, no LAI values are shown during this time. Most fish exhibited rather little change in locomotor activity during the first half of the time they were exposed to the anesthetic; however, they exhibited a marked decrease of locomotor activity during the second half. In Fish 12, this response was delayed until the fish was returned to its home tank, but then its locomotor activity was reduced for a duration comparable to the other 7 fish. In all fish, locomotor activity recovered rapidly as soon as the fish was removed from the MS-222 solution. The dotted horizontal line indicates the median of the LAIs collected at consecutive 10-s intervals during the 15 min prior to the fish’s transfer to the experimental tank containing the anesthetic, representing the individual fish’s baseline locomotor activity. The closed line indicates the smoothed LAI values obtained by using MATLAB’s *rloess* method with a span of 50 data points. Note differences in the y-axis scaling between fish

**Fig. 4:**
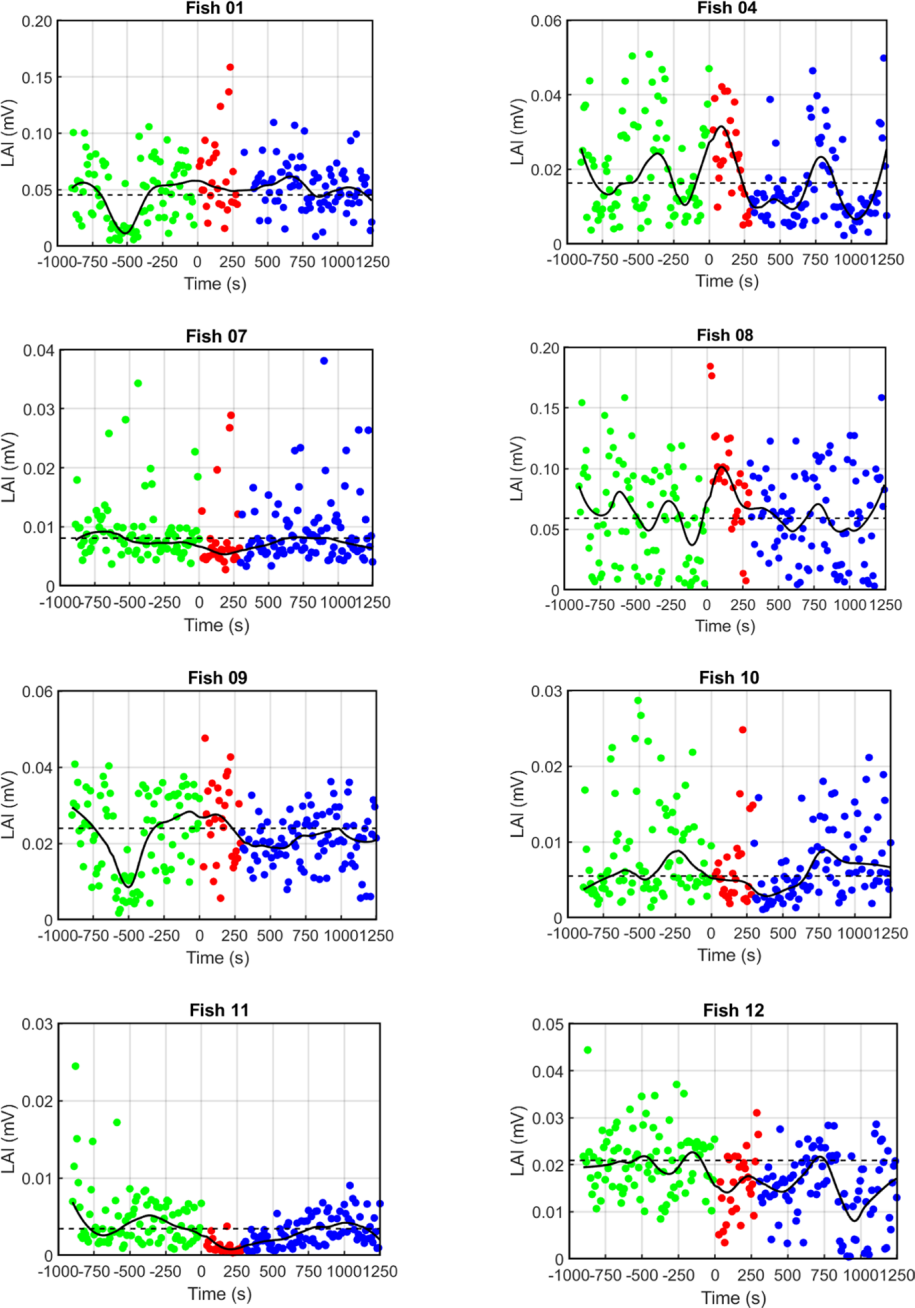
Effect of handling of fish on locomotor activity assessed by determining the LAI every 10 s. Like under test conditions, in control experiments after establishment of the baseline locomotor activity for 15 min (*green circles*), the fish was transferred from its home tank to the experimental tank containing water from the home tank only. The fish was left in this tank for 5 min (*red circles*) and then returned to its home tank to assess locomotor activity for 15 min (*blue circles*). Handling induced rather small changes in locomotor activity, which varied considerably among the 8 fish examined. The dotted horizontal line indicates the median baseline LAI. The closed line represents the smoothed LAI values. Note differences in the y-axis scaling between fish

At the beginning of each experiment, the baseline locomotor activity was determined at consecutive 10-s intervals over the 15 min immediately prior to transferring the fish to the experimental tank for conducting the anesthesia experiment or the control experiment. Visual inspection of the resulting time-LAI plots (Figs. 3, 4) revealed several features. First, the fish undergo spontaneous changes in locomotor activity, oscillating between periods of low and high LAI. Second, there is a pronounced variability among individual fish in the median baseline activity. Third, the median baseline activity of a given fish is rather constant over time, as indicated by comparing the baseline LAIs sampled in the anesthesia experiments with those collected, on different days, in the control experiments. Correlation analysis of these two data sets showed a moderate-to-strong positive correlation (Spearman rank correlation coefficient =  + 0.667, *p* = 0.071, two-tailed).

### Effect of MS-222 on locomotor activity

In each of the 8 fish examined, MS-222 induced, as expected, a reduction in locomotor activity after the fish had been transferred from its home tank to the experimental tank with the anesthetic (Figs. 1c, 3; Supplementary Information 1). In 7 of the 8 fish, this drop occurred within the 5-min exposure of the fish to the anesthetic. In Fish 12, the reduction in locomotor activity was slightly delayed and reached its maximum within the first 5 min after the fish was returned to its home tank.

To quantitatively characterize the decrease in locomotor activity, we determined the median LAI for each fish during the first 10 min following its transfer to the experimental tank (which included the fish’s 5-min exposure to the anesthetic and the following 5 min after its return to the home tank). Then, we compared these medians with the corresponding median baseline LAIs, established during the 15 min recording immediately before the fish’s transfer to the experimental tank with the anesthetic. This analysis showed that, on average, the median LAI decreased by 48% (range: decrease by 93% to increase by 28%). Statistical analysis of the medians of the 8 fish demonstrated a significant decrease in locomotor activity during the 10-min period following the transfer of the fish from the home tank to the experimental tank, compared to the corresponding baseline locomotor activity (*p* < 0.05, Related-Samples Sign Test, one-tailed, *n* = 8 fish).

To characterize the maximum drop in locomotor activity induced by the anesthesia, we calculated the median LAI in 1-min intervals over the 10-min period following the fish’s transfer from the home tank to the experimental tank and compared these values with the corresponding median baseline LAI. This analysis showed that, on average across the 8 fish, MS-222 caused the locomotor activity to drop to an average of 6% (range among the individual fish: 2–23%) of the baseline activity.

### Effect of handling of fish on locomotor activity

In the corresponding control experiments, the handling of the fish was identical to the handling in the anesthesia experiments. The only difference between the two experimental conditions was that in the control experiment the water in the experimental tank did not contain MS-222. The changes in median LAI that occurred during the 10 min following the fish’s transfer to the experimental tank with home-tank water only were less pronounced than the changes in the anesthesia experiments (Fig. 4). On average, across the 8 fish, the handling resulted in a 15% decrease in the median LAI (range: 64% decrease to 29% increase), compared to median baseline LAI. This difference was not significant (*p* > 0.05, Related-Samples Sign Test, two-tailed, *n* = 8 fish).

### Recovery of locomotor activity from MS-222 anesthesia

After the fish’s return from the experimental tank with the MS-222 solution to its home tank, recovery of the locomotor activity began within a few minutes (Fig. 3; Supplementary Information 1). In addition, as revealed by the time-versus-LAI plots, some notable differences existed between individual fish. To quantify these differences, we compared for each of the 8 fish the 60 LAIs collected during the last 10 min when the fish was back in its home tank with the median baseline LAI. The outcome of this statistical analysis is summarized in Table 1. The results show that the locomotor activity of each of the 8 fish had reached at least 50% of the corresponding baseline level, resulting in a statistically significant increase (*p* < 0.05 (Related-Samples Sign Test) of this sample. On the other hand, there was also considerable variability in recovery among the fish, as evident from the individual time-LAI plots in Fig. 3. In 3 fish, the median LAI during the last 10 min in the home tank was even higher than the corresponding median baseline LAI.

**Table 1:** Recovery of locomotor activity after anesthesia

**Fish** **number**	**Median baseline** **LAI (mV)**	**Median recovery** **LAI (mV)**	**Degree of recovery** **(%)**	***p* value** **(2-tailed)**
1	0.0548	0.0276	50	< 0.001
4	0.0212	0.0242	114	0.183
7	0.0182	0.0151	83	0.006
8	0.0393	0.0350	89	0.035
9	0.0234	0.0142	61	< 0.001
10	0.0220	0.0186	85	< 0.001
11	0.0041	0.0069	171	< 0.001
12	0.0040	0.0048	120	0.012

For each fish, the baseline LAIs were collected from the 15-min period immediately preceding the transfer of the fish from its home tank to the experimental tank containing MS-222. The 60 LAIs during the recovery period were collected from the last 10 min of the 15-min recording after the fish had been returned to its home tank. The degree of recovery was evaluated by comparing the median LAI during the recovery period (‘median recovery LAI’) with the median baseline LAI. Statistical significance was assessed with the one-sample Wilcoxon signed rank test by comparing the 60 LAIs collected during the recovery period with the corresponding median baseline LAI. The null hypothesis was that the median of the LAIs during the recovery period was not significantly different from the median baseline LAI. The *p* values are presented for a two-tailed test

### Effect of MS-222 on EOD frequency

**Fig. 5:**
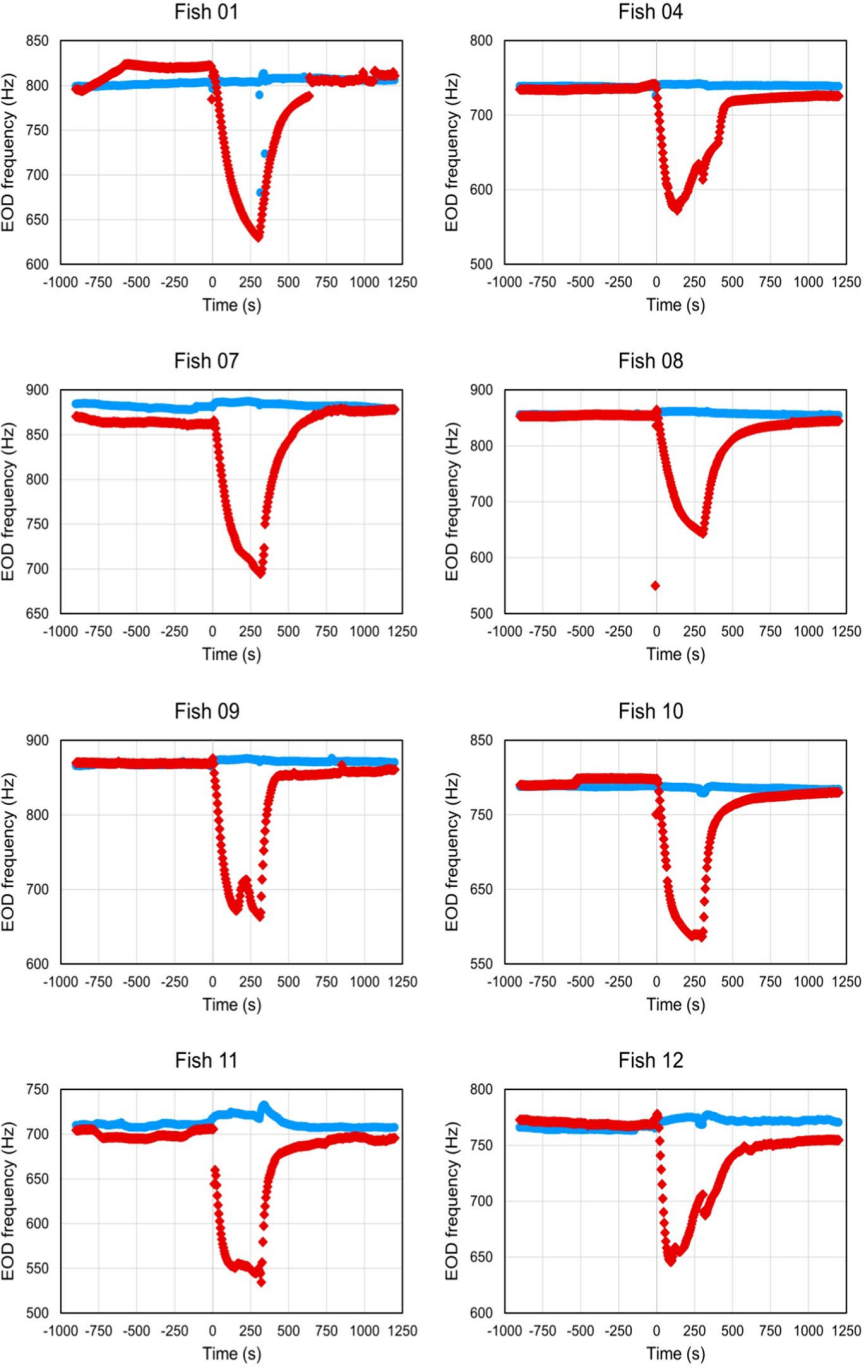
Effect of MS-222 (‘anesthesia experiment;’ *red diamonds*) and handling only (‘control experiment;’ *blue circles*) on EOD frequency. The handling of the fish was identical in the two experiments. However, in the control experiments, the experimental tank contained water from the fish’s home tank only. The plots display the temperature-adjusted frequencies in 5-s steps averaged over 10 s. In each of the 8 fish, first the baseline EOD frequency was determined in the home tank for 15 min. At time point ‘0,’ the fish were transferred to the experimental tank, where they were exposed to the anesthetic for 5 min. Then, the fish were returned to their home tanks, where EOD recording continued for 15 min. In each fish, the anesthesia induced a rapid and pronounced decrease in EOD frequency. Upon the fish’s return to the home tank, the EOD recovered largely, or even fully, within the 15-min-post-treatment period. Contrary to the fish’s exposure to MS-222, the handling of the fish under control conditions had only a minimal effect on the EOD frequency, and this effect was largely restricted to a slight increase in EOD frequency, as opposed to the dramatic decrease in EOD frequency observed in the anesthesia experiment

In each of the 8 fish, MS-222 anesthesia resulted in a fast and pronounced drop in EOD frequency (Fig. 5). During the 5th minute in the experimental tank, the median EOD frequency was 72–209 Hz (median: 172 Hz) lower than the median baseline frequency. This difference was significant at *p* = 0.008, Related-Samples Sign Test, *n* = 8 fish).

In the corresponding control experiments, transfer of the fish from their home tanks to the experimental tank containing home-tank water only caused rather minimal changes in EOD frequency, compared to baseline (Fig. 5). This included both a frequency decrease from the median baseline (in 1 fish) by 3 Hz and frequency increases from the median baseline (in 7 fish) by 3–11 Hz. The median of the frequency differences across all 8 fish during the 5th min in the experimental tank was + 5 Hz, relative to the median baseline frequency. Based on these results, we retain the null hypothesis that the median of the differences between the median baseline frequency and the median frequency in the control experiments is not statistically significantly different from 0 (*p* > 0.05, Related-Samples Sign Test, 2-sided; *n* = 8 fish).

Upon the fish’s return from the experimental tank with the MS-222 to its home tank, the EOD frequency increased — rapidly within the first few minutes, then at a slower rate. By the end of the 15-min-post-anesthesia period, the EOD frequency had either fully, or largely, recovered in each of the 8 fish.

### Effect of MS-222 on rate of chirping

**Fig. 6:**
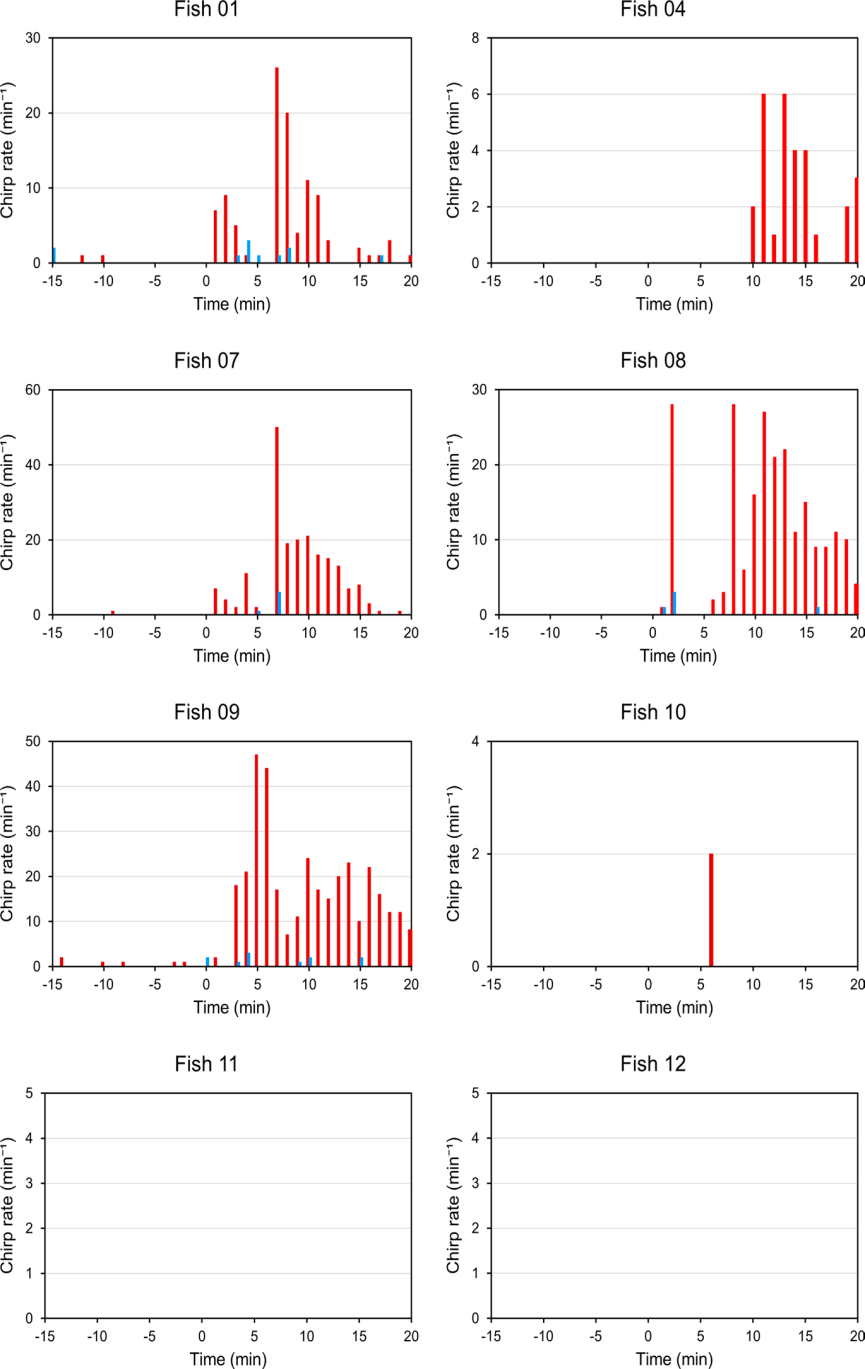
Effect of MS-222 (‘anesthesia experiment;’ *red bars*) and handling only (‘control experiment;’ *blue bars*) on chirping behavior. Chirp rates were determined during the 15 min preceding the fish’s transfer at time point 0 from the home tank to the experimental tank with the MS-222 solution (anesthesia experiment) or with water from the home tank only (control experiment), during the 5 min in the experimental tank, and the 15 min after the fish’s return to its home tank. Whereas MS-222 induced an increase in chirp rate in 6 fish, the transfers of the fish between the home tanks and the experimental tank had a rather negligible effect on chirping behavior. Note differences in y axis scaling and absence of any chirps during the EOD recordings of Fish 11 and 12

In 6 of the 8 fish examined, MS-222 anesthesia induced an increase in the rate of chirping (Fig. 6). In Fish 01, 04, 07, 08, and 09, this increase was extremely pronounced, whereas in Fish 10 the effect was very minor. Two of the 8 fish, namely Fish 11 and 12, produced chips neither during baseline recording nor during/after anesthesia. Notably, the timing of the increase in chirp rate was variable among the 6 fish. It occurred both during the 5 min of the fish’s exposure to MS-222 (in 4 fish) and/or during the 15 min following the fish’s return to their home tanks (6 fish). Moreover, in 5 of these fish the rate of chirp production remained elevated during the entire 15 min post anesthesia.

In the corresponding control experiments, 4 of the 8 fish did not generate any chirps during the entire 35 min of the EOD recording (Fig. 6). The remaining 4 fish exhibited very minor increases in chirping. Comparison of the results of the control experiments with those of the anesthesia experiments demonstratet that the increase in chirping in the 6 fish in the anesthesia experiments was due to their exposure to MS-222, rather than the transfer of the fish between their home tanks and the experimental tank.

## Discussion

In the present study, we developed an in vivo assay for the evaluation of the effect of anesthetics on locomotor activity of the weakly electric fish *A. leptorhynchus*. The quantitative analysis of this behavior is based on measurements over time of amplitude modulations of the EOD induced by changes in the position of the fish relative to the stationary external recording electrodes. As proof-of-concept of the power of this assay, we demonstrated the effect of MS-222 anesthesia on locomotor activity. At the same time, we showed that this locomotor activity assay can be integrated into our previously developed bimodal Neuro-Behavioral Assay, using the same EOD recording. Taken together, this combined, trimodal assay offers the possibility to evaluate differential effects of anesthetics simultaneously on three behaviors: locomotor activity, EOD frequency, and chirping behavior.

### Gauging locomotor activity through changes in EOD amplitude

The locomotor activity assay is grounded on the physical properties of the EOD. In *A. leptorhynchus*, the EOD is generated by a neurogenic electric organ in the trunk, composed of enormously enlarged axonal terminals of the electromotor neurons. These so-called electrocytes are stacked in rows in the trunk, where they discharge nearly synchronously. The resulting compound action potential can be detected non-invasively by recording with external electrodes (Fig. 1a) (for review see Zupanc and Bullock 2005).

At distances greater than the fish’s size, the spatial geometry of its electric field approximates that of an ideal dipole, i.e., it is a vector field (for a review of the physics of the bioelectric fields generated by electric fish see Benda 2020). Thus, the field strength at each point in space is defined by a vector with length and direction. The magnitude of this dipole field is proportional to the increase of distance cubed — doubling the distance leads to an eightfold drop in EOD amplitude. In other words, changes of the fish’s orientation to, and/or distance from, the recording electrodes are reflected by pronounced modulations of the *recorded* EOD amplitude (while there is no evidence that the amplitude of the *emitted* EOD changes over the time intervals used in our experiments). This property makes it possible to assess relative changes in locomotor activity manifested by alteration of distance and/or orientation of the fish through evaluation of the relative changes in EOD amplitude over time.

As a reflection of these relative changes, we defined the LAI by the degree of variability of the recorded amplitudes over time. Note that changes in locomotor activity of a fish are reflected by proportional, but not linear, changes in its LAIs. The LAI is best suited as an indicator of the relative differences in locomotor activity, recorded in the same individual fish at different points in time. Due to possible differences in the electric fields generated by different fish (caused, among others, by differences in the size of the individuals), LAIs collected from different fish cannot be directly compared with each other.

### Evaluation of the effect of anesthetics on three different behavioral outcomes

A unique feature of the updated Neuro-Behavioral Assay is its capability to evaluate the effect of anesthetics on three different behavioral outcomes (locomotor activity, EOD frequency, and chirping behavior), yet the analysis of each of these effects is based on data extracted from the same recorded behavior (the EOD) obtained non-invasively with minimal restraint of the animal. The simultaneous recording of these behaviors, in a single trace, greatly facilitates direct comparison of the time course of these behavioral patterns, and thus exploration of possible differential effects of a given anesthetic on different behaviors and the physiological activity of the underlying different neural networks.

In the present study, we have demonstrated such a differential effect of MS-222. Whereas this anesthetic induced, in each of the 8 fish examined, dramatic changes in LAI and EOD frequency within a few minutes, an increase in chirp rate (i) was observed only in some but not all fish; (ii) occurred with notable inter-individual variability in both the degree and the time course of the alteration of the behavior.

Similarly, the fish displayed different times courses of recovery. While most (but not all) of the fish regained locomotor activity within 15 min post exposure to MS-222, neither EOD frequency nor chirp rate had fully recovered within this time frame. Previously, we had studied specifically recovery of the latter two behaviors using the same concentration of MS-222 and similar experimental conditions as in the present investigation (Eske et al. 2023). We found that the decrease in EOD frequency can sustain for up to 1 h, and that chirp rates remain elevated, although at rather low levels, up to approximately 3 h. Taken together, the results of the current investigation and the study by Eske et al. (2023) underscore the need to examine the effect of a given anesthetic on more than one behavior in order to gain at least a basic understanding of the complexity of its neuromodulatory potential.

At the same time, it is important to pay attention to inter-individual variability of the effects of anesthetics (like of other drugs) due to genomic/phenotypic diversity, an aspect increasingly being recognized in drug discovery (Harrill et al. 2009; French et al. 2015; Gatti et al. 2018; Dumont et al. 2024). The foundation for studying such diversity is provided using wild-type fish in the Neurobehavioral Assay, and its ability to resolve fine details of the fish’s behavioral response to a treatment regime.

### Capturing the continuous nature of behavioral responses

Another significant advantage of the Neuro-Behavioral Assay is that the effects of anesthetics on locomotor activity, EOD frequency, and chirp rate are quantified on a continuous scale. Thus, the time-dependent data obtained through these measurements reflect well the fact that organisms, including humans, reach the various stages of anesthesia gradually. By contrast, other in vivo assays established over the last three decades generate data based on an (often arbitrarily defined) binary outcome of a behavioral response. Examples are the ability or the failure of tadpoles and adult frogs (American bullfrog, *Rana catesbeiana*; *X. laevis*) to right themselves from a supine position (‘righting reflex assay’) (Downes and Courogen 1996; Krasowski et al. 2001); the presence or absence of a startle response evoked by a sharp tap on the lid of a Petri dish in which a *Xenopus* tadpole is placed (‘startle response assay’) (McKinstry-Wu et al. 2015); and the occurrence (or lack thereof) in response to a light flash of a spike in motor activity exhibited by zebrafish larvae kept in a dark chamber (‘photomotor response assay’) (Yang et al. 2018). The use of binary endpoints of the behavioral responses in these assays makes it difficult, if not impossible, to determine the exact time course of induction of, and/or recovery from, anesthesia. For example, according to the protocol used in the *Xenopus* larvae startle response assay, tadpoles are incubated with solutions of the anesthetic compound for 1 h and then scored for the behavioral response — the lack of a startle response (McKinstry-Wu et al. 2015). By contrast, the LAI in the Neuro-Behavioral Assay provides detailed information on a time scale with second resolution about the fish’s gradual transition from normal locomotor activity to the complete cessation of any movement. Similarly, the time course of recovery from the anesthesia can be fully reconstructed using the LAI data. This is a significant advantage, as it reflects more adequately the gradual transition of an organism to the different stages of anesthesia, and the subsequent gradual recovery from these stages. The same advantage applies to the other two behavioral parameters quantified by the Neuro-Behavioral Assay, the EOD frequency and the chirp rate.

## Conclusion

The Neuro-Behavioral Assay developed during this investigation and previously (Eske et al. 2023; Lehotzky et al. 2023) provides a powerful tool for the evaluation of behavioral and neural effects of anesthetics. It is the only in vivo assay currently available that employs simultaneously three behavioral paradigms to assess these effects: locomotor activity, EOD frequency, and chirping behavior. While locomotor activity is one of the most obvious behaviors affected by anesthesia, it has been rather surprising that each of the three anesthetics examined thus far (MS-222: Eske et al. 2023, this study; urethane: Eske et al. 2023; eugenol: Lehotzky et al. 2023) induces a fast and pronounced drop in EOD frequency. At the same time, this highly robust effect has offered the unique opportunity to assess the effect of these (and likely other) anesthetics on the physiological function of a CNS structure because the frequency of the EOD can be used as a proxy of the frequency of an endogenous oscillator, the pacemaker nucleus, in the brainstem. This is possible because the neural command signals from the pacemaker nucleus drive the discharges of the electric organ in a one-to-one fashion (for review see Zupanc and Bullock 2005). Thus, the effects of anesthetics on the oscillation frequency of the pacemaker nucleus can be assessed non-invasively through recordings of the EOD. Chirps, on the other hand, reflect modulations of the normal oscillation pattern of the pacemaker nucleus by input from other brain regions (for review see Metzner 1999). The combination of these three neuro-behavioral paradigms offers the option to study a frequently neglected but important aspect — the phenomenon that different CNS systems may be differentially affected by a given anesthetic.

Taken together, the rich, quantitative information provided by the Neuro-Behavioral Assay offers an unprecedented degree of depth of evaluation of the on-target and off-target effects of anesthetics.

## Supplementary Information

Below is the link to the electronic supplementary material.

Supplementary file1 (MOV 317613 KB)

## Data Availability

Data that support the findings of this study are available from the corresponding author upon reasonable request.
